# Indication for spinal sensitization in chronic low back pain: mechanical hyperalgesia adjacent to but not within the most painful body area

**DOI:** 10.1097/PR9.0000000000001166

**Published:** 2024-06-17

**Authors:** Laura Sirucek, Iara De Schoenmacker, Paulina Simonne Scheuren, Robin Lütolf, Lindsay Mary Gorrell, Anke Langenfeld, Mirjam Baechler, Jan Rosner, Brigitte Wirth, Michèle Hubli, Petra Schweinhardt

**Affiliations:** a Department of Chiropractic Medicine, Balgrist University Hospital, University of Zurich, Zurich, Switzerland; b Neuroscience Center Zurich, University of Zurich, Zurich, Switzerland; c Spinal Cord Injury Center, Balgrist University Hospital, University of Zurich, Zurich, Switzerland; d Biomedical Data Science Lab, Institute of Translational Medicine, Swiss Federal Institute of Technology (ETH) Zurich, Zurich, Switzerland; e International Collaboration on Repair Discoveries (ICORD), University of British Columbia, Vancouver, BC, Canada; f Department of Neurology, University Hospital Bern, Inselspital, University of Bern, Bern, Switzerland; g Danish Pain Research Center, Department of Clinical Medicine, Aarhus University, Aarhus, Denmark

**Keywords:** Quantitative sensory testing, Chronic pain, Central sensitization, Secondary hyperalgesia

## Abstract

**Introduction:**

In 85% of patients with chronic low back pain (CLBP), no specific pathoanatomical cause can be identified. Besides primary peripheral drivers within the lower back, spinal or supraspinal sensitization processes might contribute to the patients' pain.

**Objectives:**

The present study conceptualized the most painful area (MP) of patients with nonspecific CLBP as primarily affected area and assessed signs of peripheral, spinal, and supraspinal sensitization using quantitative sensory testing (QST) in MP, a pain-free area adjacent to MP (AD), and a remote, pain-free control area (CON).

**Methods:**

Fifty-nine patients with CLBP (51 years, SD = 16.6, 22 female patients) and 35 pain-free control participants individually matched for age, sex, and testing areas (49 years, SD = 17.5, 19 female participants) underwent a full QST protocol in MP and a reduced QST protocol assessing sensory gain in AD and CON. Quantitative sensory testing measures, except paradoxical heat sensations and dynamic mechanical allodynia (DMA), were *Z*-transformed to the matched control participants and tested for significance using *Z*-tests (α = 0.001). Paradoxical heat sensations and DMA occurrence were compared between cohorts using Fisher's exact tests (α = 0.05). The same analyses were performed with a high-pain and a low-pain CLBP subsample (50% quantile).

**Results:**

Patients showed cold and vibration hypoesthesia in MP (all *P*s < 0.001) and mechanical hyperalgesia (*P* < 0.001) and more frequent DMA (*P* = 0.044) in AD. The results were mainly driven by the high-pain CLBP subsample. In CON, no sensory alterations were observed.

**Conclusion:**

Mechanical hyperalgesia and DMA adjacent to but not within MP, the supposedly primarily affected area, might reflect secondary hyperalgesia originating from spinal sensitization in patients with CLBP.

## Abstract 2

Quantitative sensory testing in 3 different body areas implied a potential involvement of spinal sensitization in patients with chronic low back pain.

## 1. Introduction

Chronic low back pain (CLBP) is a particularly challenging chronic pain condition because in approximately 85% of cases, no specific pathoanatomical cause can be identified,^35^ which hinders mechanism-based treatment approaches. Various lumbar structures such as facet joints^57^ or intervertebral disks^29^ might contribute to CLBP, as well as central processes, ie, central sensitization.^3,22,49^ Different definitions of central sensitization exist,^13^ including the originally described activity-dependent central sensitization at the spinal dorsal horn neuron^65^ and the broader definition of the International Association for the Study of Pain, that is “increased responsiveness of nociceptive neurons in the central nervous system to their normal or subthreshold afferent input.”^28^ Although it is not possible to directly measure neuronal hyperexcitability, and thus central sensitization, in humans, certain sensory signs serve as proxies.^3^ Quantitative sensory testing (QST)^44^ allows the standardized assessment of such sensory signs, for example, allodynia, hyperalgesia, or increased temporal summation of pain.^3^ Of note, to infer a central—and not peripheral—origin of the respective sensory signs, QST has to be performed in more than one body area.^3^ For instance, dynamic mechanical allodynia (DMA) and mechanical hyperalgesia are hallmarks of spinal sensitization as demonstrated in experimentally induced activity-dependent central sensitization,^31,48,56^ but only if present in a secondary area, ie, a region surrounding the primarily affected area. If detected in the primarily affected area, DMA and mechanical hyperalgesia could be due to peripheral or spinal sensitization.^3^ In addition, supraspinal sensitization may contribute to DMA, mechanical hyperalgesia, and other signs of pain hypersensitivity in any body area given that alterations in supraspinal neuronal circuits can have widespread effects, for example in the case of dysfunctional descending pain inhibition.^5^ One option to differentiate supraspinal from spinal or peripheral sensitization is to assess pain hypersensitivity at body areas remote from the primarily affected or the secondary area.^3^

The present study aimed to apply these concepts in patients with nonspecific CLBP and pain-free control participants. The most painful area (MP) within the lower back of patients with CLBP was considered a proxy for the primarily affected area. A pain-free area adjacent to MP (AD) was conceptualized as secondary area surrounding the primarily affected area. The pain-free nondominant hand served as remote, pain-free control area (CON). In these 3 body areas, sensory alterations were examined using the QST battery provided by the German Research Network on Neuropathic Pain (DFNS)^44^ to infer a putative presence of peripheral, spinal, and supraspinal sensitization in the patient cohort. In addition, it was explored whether QST measures indicative of spinal or supraspinal sensitization were related to patient-reported outcome measures supposedly associated with central pain processes, namely pain catastrophizing,^7,54,59^ sleep quality,^7^ and widespread pain.^42^

## 2. Methods

### 2.1. Participants

Patients with nonspecific CLBP and individually age- and sex-matched pain-free control participants between 18 and 80 years of age were consecutively recruited through the Balgrist University Hospital, online advertisement, and oral communication. Patients with CLBP needed to present with CLBP as primary pain complaint without signs of serious underlying pathology (eg, infection, fractures, or inflammation) or radiculopathy (ie, motor and sensory deficits) and of a duration longer than 3 months. Control participants could not have experienced low back pain lasting longer than 3 consecutive days during the last year. Exclusion criteria comprised any self-reported major medical (eg, severe heart disease) or psychiatric (eg, major depressive disorder) condition other than CLBP, pregnancy, or inability to follow study instructions. Ethical approval was obtained from the local ethics committee “Kantonale Ethikkommission Zürich” (Nr.: PB_2019-00136, PB_2016-02051, and EK-04/2006). The study was registered on ClinicalTrials.gov (NCT04433299 and NCT02138344) and performed in accordance with the Declaration of Helsinki (2013). All participants provided written informed consent before the start of the experiment.

### 2.2. Quantitative sensory testing

The QST battery was part of a larger study protocol (Clinical Research Priority Program “Pain”, https://www.crpp-pain.uzh.ch/en.html) that comprised 3 sessions of approximately 3 hours and electronic questionnaires, including the Hospital Anxiety and Depression Scale (HADS; anxiety and depression subscales, each scored from 0 to 21, with higher scores meaning greater anxiety or depression),^66^ the Pain Catastrophizing Scale (PCS; scored from 0 to 52, with higher scores meaning more pronounced pain catastrophizing),^52^ the Pain and Sleep Questionnaire 3-item index (PSQ-3; scored from 0 to 300, with higher scores meaning more severe pain-related sleep disturbances),^4^ the painDETECT (scored from 0 to 38, with higher scores indicating a more likely neuropathic pain component),^17^ and the Widespread Pain Index (WPI; scored from 0 to 19, with higher scores indicating a larger number of painful body regions).^64^ The electronic questionnaires also included a question about regular pain-relevant medication intake that was classified into M01A (anti-inflammatory and anti-rheumatic drugs and nonsteroids), N02 (analgesics), N03 (antiepileptics), N05 (psycholeptics), and N06 (psychoanaleptics) based on the ATC/DDD classification by the World Health Organization (http://www.whocc.no/atc_ddd_index/). Quantitative sensory testing was performed in the first session after a clinical examination (for details see section 2.3) and a neurophysiological assessment.

**Figure 1.**
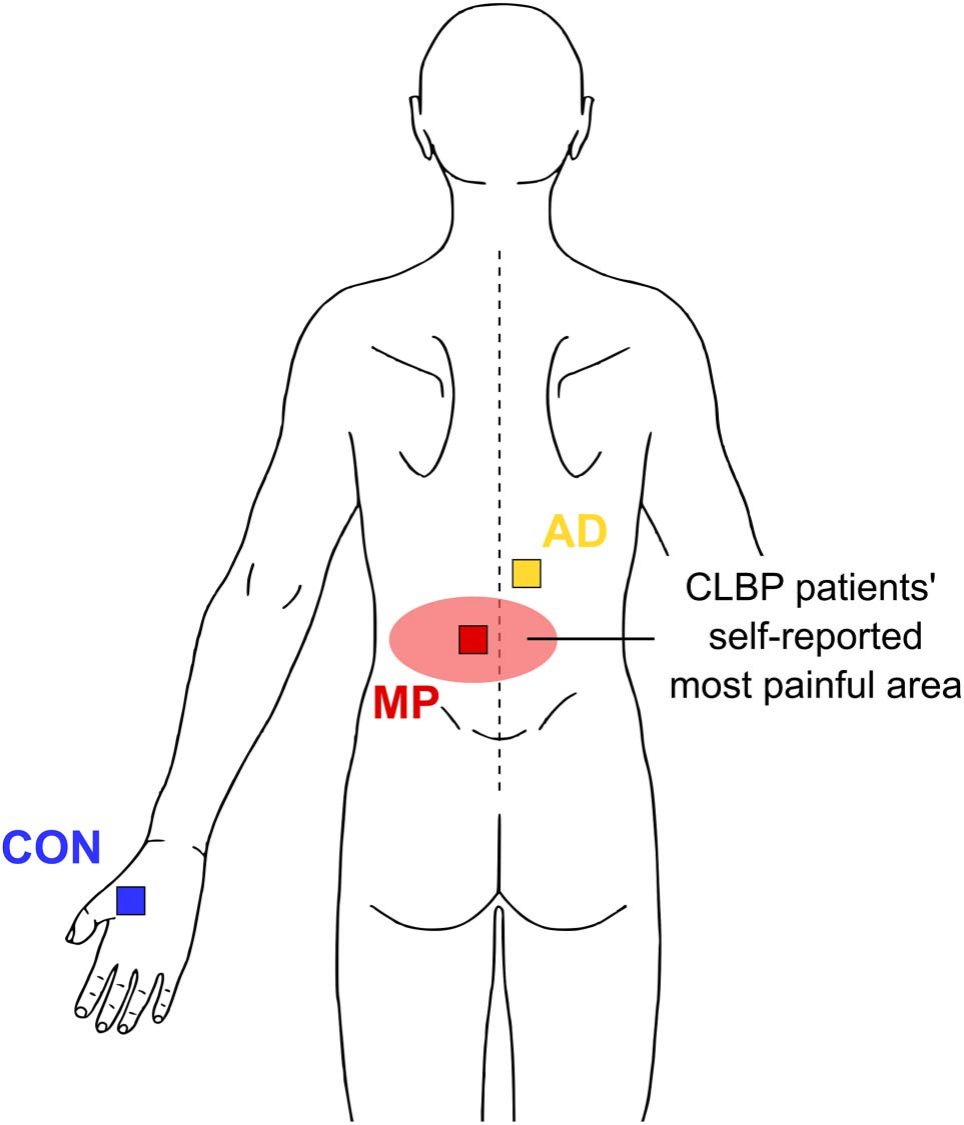
Schematic illustration of QST testing areas. For the most painful area (MP), superficial QST measures were assessed in the centre of MP, PPT was assessed over the erector spinae muscle at the segmental level of MP and VDT was assessed over the processus spinosus at the segmental level of MP. For the pain-free area adjacent to MP (AD), all QST measures were assessed over the erector spinae muscle (contralaterally to MP if MP was lateralized). For the remote, pain-free control area (CON), the standard DFNS locations were used, ie, dorsum of the hand for superficial QST measures and the thenar eminence for PPT. DFNS, German Research Network on Neuropathic Pain; PPT, pressure pain thresholds; QST, quantitative sensory testing; VDT, vibration detection thresholds.

During the clinical examination, the location of the patients' MP within the lower back was identified. In addition, the patients were asked to indicate the area closest to MP (rostrally), which they perceived as pain-free (AD). If the patients' MP was lateralized, AD was assessed at the contralateral body side to avoid potential confounding of QST measures by tension in the erector spinae muscle. If the patients' MP was in the midline of the back, the body side to assess AD was randomly chosen. The nondominant hand served as the remote, pain-free control area (CON) (Fig. 1). For CON, normal sensory integrity was tested before the QST session for all participants by bedside sensory testing of vibration, thermal, pinprick, and light touch sensation. Control participants were assessed at the identical testing sites as the patient with CLBP they had been matched to.

In MP, the full DFNS QST protocol^44^ was performed to assess sensory loss and gain of function. In AD and CON, a reduced QST protocol focusing on the sensory gain of interest was performed. The reduced protocol comprised the following: cold pain thresholds (CPT), heat pain thresholds (HPT), mechanical pain thresholds (MPT), mechanical pain sensitivity (MPS), DMA, wind-up ratio (WUR), and pressure pain thresholds (PPT). Because of time constraints within the larger study protocol, MPS and DMA were evaluated with 2 (instead of 5) stimulus-response-function blocks. Pilot measurements in our laboratory showed that MPS and DMA values depend on the number of included blocks, and therefore, MPS and DMA were calculated based on the first 2 stimulus-response-function blocks also in MP to ensure comparability across all areas. Based on DFNS recommendations, CON was always assessed first. Given that the applied QST protocol deviated from the full DFNS QST protocol, a comparison of the CLBP patients' QST measures to the DFNS reference database would not have been valid. Therefore, the control participants who underwent the identical QST protocol as the patients with CLBP were used as reference group.

All QST measures except paradoxical heat sensations (PHS) and DMA were *Z*-transformed to the control participants. For that, control participants were divided into age-based tertiles and values of patients with CLBP were *Z*-transformed to the control participant tertile of their age. This allowed for calculation of *Z*-scores referenced to identical body areas, which would not have been possible with the DFNS reference database. To avoid the influence of single extreme outliers on the analysis, the maximum and minimum *Z*-score value was set to ±4.^11^

Quantitative sensory testing changes in patients with CLBP were tested by comparing the *Z*-values of patients with CLBP to an assumed ideal healthy population (ie, an ideal *Z*-value distribution with mean = 0 and SD = 1) using *Z*-tests with a conservative α = 0.001 to reduce the risk for false-positive results.^53^ Fisher's exact tests with α = 0.05 were used to compare the presence or absence of PHS and DMA between the cohorts. False discovery rate (FDR) multiple comparison correction for 3 tests was performed for those QST measures that were assessed on all 3 body areas. As an additional exploratory analysis, the same approach was performed with the patients with CLBP divided into a high-pain and a low-pain subsample (based on 50% quantile).

Associations between QST measures indicative of spinal (mechanical sensory gain of function in AD, ie, MPT, PPT, MPS, and WUR) or supraspinal sensitization (thermal and mechanical sensory gain of function in CON, ie, CPT, HPT, MPT, PPT, MPS, and WUR) and patient-reported outcome measures supposedly associated with central pain processes (ie, PCS, PSQ-3, and WPI) were investigated using Spearman correlations. Because of the exploratory nature of these correlation analyses, no multiple comparison correction was performed.

### 2.3. Clinical examination

The clinical examination was based on an evidence-based diagnostic classification system for low back pain^60,61^ and comprised diagnostic tests to investigate the most likely underlying nociceptive source of the patients' nonspecific CLBP, for example, provocation tests for discogenic/facetogenic/sacroiliac (ie, nociceptive) or radicular (ie, neuropathic) pain. Together with the painDETECT and the WPI, this information was used to characterize the patient cohort in potential underlying nociceptive, neuropathic, or nociplastic pain mechanisms.^51^ Based on the Delphi consensus study, the following features were considered to be indicative of nociceptive pain mechanisms: clear discogenic/facetogenic/sacroiliac symptom provocation pattern, painDETECT scores ≤12, and localized pain extent. Localized pain extent was inferred if patients with CLBP did not meet the criteria of widespread (contralateral limb) pain assessed using the WPI.^23,64^

## 3. Results

### 3.1. Participants

From the recruited 64 patients with CLBP and 48 control participants, 8 participants were excluded because of discontinuation (3 patients), abnormal sensory findings (2 control participants), suspected neurological (1 patient) or psychiatric (1 patient) conditions, or development of low back pain between the time of inclusion and the experimental session (1 control participant). Thus, the final sample comprised 59 patients with CLBP and 45 control participants. Participant characteristics are described in Table 1. Ten of the 45 control participants had been matched to another chronic pain cohort of the Clinical Research Priority Program “Pain” for MP and AD and were therefore only included in analyses related to CON.

**Table 1:** Participant characteristics.

	Patients with CLBP (n = 59)	Control participants (n = 45)	Test statistic	*P*	Effect size
Age [y]	50.8 (16.64)	48.1 (16.85)	*t* = 0.8	0.412	*d* = 0.16
Sex (female:male) [n]	37:22	26:19		0.687*	
BMI [kg/m^2^]	23.9 (3.47)	23.4 (2.87)	*t* = 0.8	0.422	*d* = 0.16
HADS anxiety	4 (2.5–7.0)	3 (2.0–5.0)	***W* = 789.0**	**0.036**	***r* = 0.22**
HADS depression	3 (1.0–6.0)	1 (0–2.0)	***W* = 594.5**	**<0.001**	***r* = 0.37**
PCS	10 (4.0–21.0)	2 (0–8.0)	***W* = 484.0**	**<0.001**	***r* = 0.46**
CLBP characteristics					
Pain characteristics					
Clinical pain intensity [NRS]†	4 (3.0–5.0)				
Pain duration [mo]	79 (15.5–202.3)‡				
Spatial pain extent [%]§	1.3 (0.55–2.30)				
PSQ-3	84 (25.0–127.5)				
WPI	4 (2.0–6.0)				
Nociceptive pain features					
Clear symptom provocation pattern (n/%)	37/62.7				
painDETECT ≤ 12 (n/%)	47/82.5‖				
Localized pain (n/%)	56/94.9				
Myofascial features¶					
Myofascial component (n/%)	50/84.7				

High-/low-pain CLBP subsamples					
	High-pain (n = 32)	Low-pain (n = 27)			
Age [y]	48.0 (16.90)	54.2 (16.00)	*t* = 1.4	0.157	*d* = −0.37
Sex (female:male) [n]	19:13	18:9		0.600	
BMI [kg/m^2^]	24.0 (3.24)	23.8 (3.78)	*t* = 0.2	0.811	*d* = 0.06
HADS anxiety	5 (2.8–7.3)	4 (2.5–6.5)	*W* = 463.0	0.641	*r* = 0.06
HADS depression	3 (1.0–6.0)	2 (2.0–6.0)	*W* = 421.5	0.878	*r* = 0.02
PCS	11 (4.8–23.3)	10 (3.5–15.0)	*W* = 515.0	0.209	*r* = 0.16
Clinical pain intensity [NRS]†	**5 (4.0–6.0)**	**3 (2.0–3.0)**	***W* = 864.0**	**<0.001**	***r* = 0.87**
Pain duration [mo]	39 (12.0–142.5)	135 (22.5–260.0)	*W* = 301.5	0.069	*r* = 0.24
Spatial pain extent [%]§	1.4 (0.68–2.25)	1.1 (0.40–2.00)	*W* = 501.0	0.297	*r* = 0.14
PSQ-3	**99 (74.5–148.8)**	**35 (11.0–92.0)**	***W* = 635.5**	**0.002**	***r* = 0.40**
WPI	4 (3.0–6.0)	4 (1.0–6.0)	*W* = 461.0	0.662	*r* = 0.06

High-pain (NRS ≥ 4/10) and low-pain (NRS < 4/10) CLBP subsamples were formed based on the 50% quantile of clinical pain intensities. Values are presented as mean (SD) for continuous variables and as median (interquartile range) for ordinal or nonnormally distributed variables. *T*-statistics refer to unpaired *t*-tests and *W*-statistics to Wilcoxon rank sum tests. Effect sizes are reported as Cohen's *d* (small: < 0.5, medium: 0.5–0.8, large: > 0.8)^9^ for *t*-tests and *r* (small: 0.1–< 0.3, medium: 0.3–< 0.5, large: ≥ 0.5)^10^ for Wilcoxon rank sum tests.

* Fisher's exact test.

† Average clinical pain intensity over the past 4 weeks, self-reported through electronic questionnaires completed before the QST session.

‡ N = 40 because of 1 missing value (participant did not indicate month of pain onset).

§ Extent of typically painful LBP-associated body areas (in % of total body area), ie, the lower back, the buttocks and the legs, were assessed using pain drawings.^45^

‖ Proportion relative to 57 patients with CLBP because of 2 missing values in the painDETECT.

¶ Based on the clinical examination.

BMI, body mass index; CLBP, chronic low back pain; HADS, Hospital Anxiety and Depression Scale; NRS, numeric rating scale; PCS, Pain Catastrophizing Scale; PSQ-3, Pain and Sleep Questionnaire three-item index; WPI, Widespread Pain Index. Bold entries: *P* < 0.05.

### 3.2. Quantitative sensory testing

**Figure 2.**
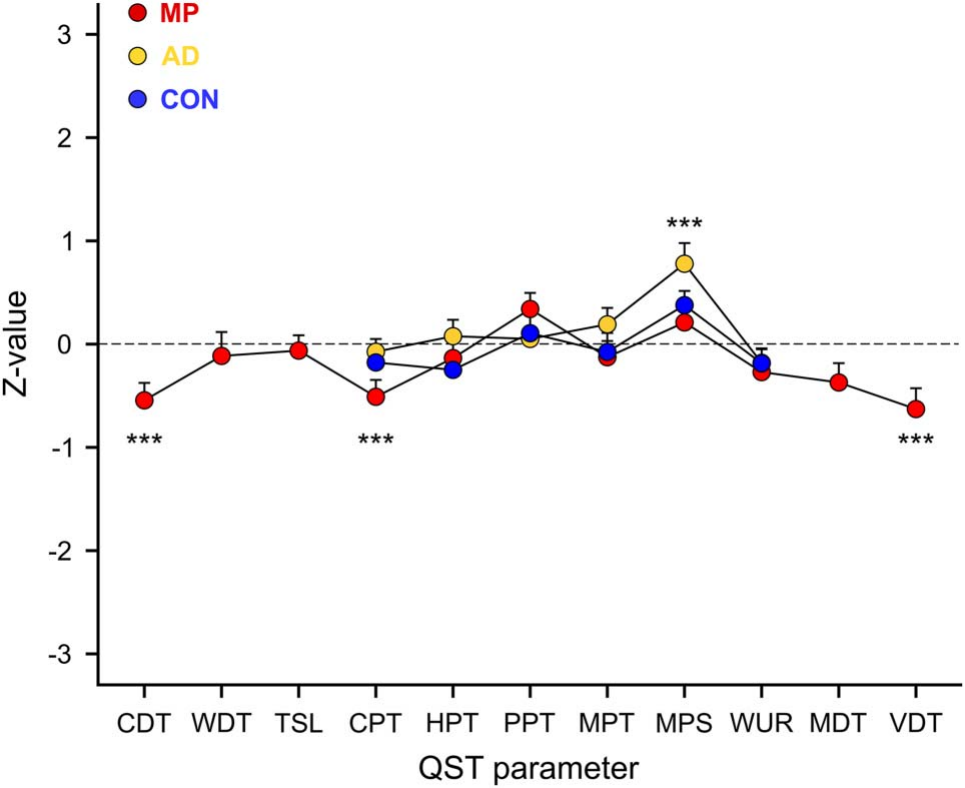
Patients with CLBP show sensory loss of function in MP and sensory gain of function in AD. Quantitative sensory testing (QST) profiles of the 3 tested areas, ie, most painful area (MP), pain-free area adjacent to MP (AD), and remote, pain-free control area (CON). Data are presented as mean *Z*-values and SEs. CDT, cold detection threshold; CLBP, chronic low back pain; CPT, cold pain threshold; HPT, heat pain threshold; MDT, mechanical detection threshold; MPS, mechanical pain sensitivity; MPT, mechanical pain threshold; PPT, pressure pain threshold; TSL, thermal sensory limen; VDT, vibration detection threshold; WDT, warm detection threshold; WUR, wind-up ratio. ***P < 0.001.

In MP, patients with CLBP presented with increased cold detection thresholds (CDT) in combination with decreased cold pain thresholds (CPT) and vibration detection thresholds (VDT), indicating sensory loss of function (Table 2, Fig. 2). The pain-free area adjacent to MP was located contralaterally to MP in 44 of 59 patients with lateralized MP and randomly assigned to a body side in 15 of 59 patients with MP in the midline of the back. In AD, patients with CLBP showed increased MPS and more frequent DMA compared with control participants, reflecting sensory gain of function (Table 2, Fig. 2). In all remaining QST measures, as well as in CON, patients with CLBP showed neither sensory gain nor loss of function (Table 2, Fig. 2). Fifteen extreme *Z*-scores (ie, >4 or < −4) were identified and adjusted to 4 or −4, respectively (MP > 4: 1 warm detection threshold (WDT), 1 DMA; MP < −4: 2 CDT, 2 WDT, 1 mechanical detection threshold (MDT), 4 VDT; AD > 4: 1 MPS, 1 DMA; AD < −4: 1 MPT; CON > 4: 1 WUR).

**Table 2:** Quantitative sensory testing measures in patients with chronic low back pain and control participants.

	Patients with CLBP* (n = 59)	Control participants* (n = 45)	Patients with CLBP *Z*-score	*Z* statistic	*P*
MP†					
CDT [°C]	**3.0 (2.14)**	**2.3 (1.39)**	**−0.5 (1.30)**	**−4.2**	**<0.001**
WDT [°C]	3.3 (1.49)	3.0 (0.84)	−0.1 (1.78)	−0.9	0.380
TSL [°C]	6.8 (3.02)	6.5 (2.13)	−0.1 (1.14)	−0.5	0.629
CPT [°C]	**11.9 (10.35)**	**15.9 (10.22)**	**−0.5 (1.25)**	**−3.9**	**<0.001‡**
HPT [°C]	43.1 (3.66)	42.7 (3.46)	−0.1 (1.10)	−1.0	0.447‡
PPT [kg/cm^2^]	5.3 (2.66)	6.0 (2.45)	0.3 (1.20)	2.6	0.027‡
MPT [mN]	33.4 (36.80)	29.4 (30.56)	−0.1 (1.20)	−1.0	0.495‡
MPS [NRS]	4.8 (5.46)	3.8 (4.33)	0.2 (1.08)	1.6	0.107‡
WUR [NRS ratio]	3.0 (1.98)	3.6 (2.25)	−0.3 (0.93)	−2.1	0.112‡
MDT [mN]	16.9 (31.68)	7.6 (6.01)	−0.4 (1.44)	−2.9	0.004
VDT [V.U.]	**5.5 (2.62)**	**6.5 (1.35)**	**−0.6 (1.55)**	**−4.8**	**<0.001**
PHS [count]	2	3			0.357§
DMA [count]	13	6			0.608‡§
AD†					
CPT [°C]	10.4 (10.67)	11.6 (11.59)	−0.1 (0.95)	−0.6	0.572‡
HPT [°C]	43.3 (3.79)	43.4 (3.12)	0.1 (1.22)	0.6	0.557‡
PPT [kg/cm^2^]	5.8 (2.40)	5.9 (2.46)	0.1 (0.88)	0.4	0.699‡
MPT [mN]	45.2 (67.95)	55.2 (83.74)	0.2 (1.22)	1.5	0.425‡
MPS [NRS]	**4.0 (3.96)**	**1.6 (1.29)**	**0.8 (1.54)**	**6.0**	**<0.001‡**
WUR [NRS ratio]‖	3.1 (2.04)	3.2 (1.82)	−0.2 (1.07)	−1.4	0.167‡
DMA [count]	**13**	**1**			**0.044** ‡ §
CON					
CPT [°C]	8.9 (9.32)	10.6 (7.75)	−0.2 (1.15)	−1.4	0.258‡
HPT [°C]	45.1 (3.64)	44.4 (3.34)	−0.2 (1.09)	−1.9	0.167‡
PPT [kg/cm^2^]	4.3 (2.06)	4.2 (1.64)	0.1 (1.34)	0.8	0.626‡
MPT [mN]	54.5 (69.08)	40.6 (33.2)	−0.1 (1.41)	−0.6	0.561‡
MPS [NRS]	4.7 (5.64)	2.9 (3.31)	0.4 (1.06)	2.9	0.006‡
WUR [NRS ratio]	3.4 (8.38)	2.8 (1.72)	−0.2 (1.05)	−1.4	0.167‡
DMA [count]	10	3			0.215‡§

All QST measures except for CPT, HPT, and VDT were log-transformed before *Z*-score conversion. Values are presented as mean (SD). Minimum and maximum *Z*-score value for each QST measure was set to ±4 to reduce influences of single extreme outliers. Effect sizes are not reported because for *Z*-tests against mean = 0 and SD = 1, Cohen's *d* is identical to the mean of the *Z*-scores.

* Raw values might not reflect *Z*-scores because (1) most QST measures were log-transformed before *Z*-score conversion, (2) outlier influences are larger for raw values than for *Z*-scores, and (3) raw values are not age and sex matched.

† N = 35 for control participants because 10 control participants had been matched to another chronic pain cohort of the Clinical Research Priority Program “Pain.”

‡ FDR-corrected for 3 tests.

§ Fisher's exact test.

‖ N = 58 for patients with CLBP because 1 patient rated the highest possible single stimulus intensity as not painful.

AD, pain-free area adjacent to most painful area; CDT, cold detection threshold; CLBP, chronic low back pain; CON, remote, pain-free control area; CPT, cold pain threshold; DMA, dynamic mechanical allodynia; FDR, false discovery rate; HPT, heat pain threshold; MDT, mechanical detection threshold; MPS, mechanical pain sensitivity; MPT, mechanical pain threshold; NRS, numeric rating scale; PHS, paradoxical heat sensation; PPT, pressure pain threshold; QST, quantitative sensory testing; TSL, thermal sensory limen; VDT, vibration detection thresholds; V.U., vibration units in X/8; WUR, wind-up ratio. Bold entries: *P* < 0.05 for PHS and DMA and *P* < 0.001 for other QST measures.

**Figure 3.**
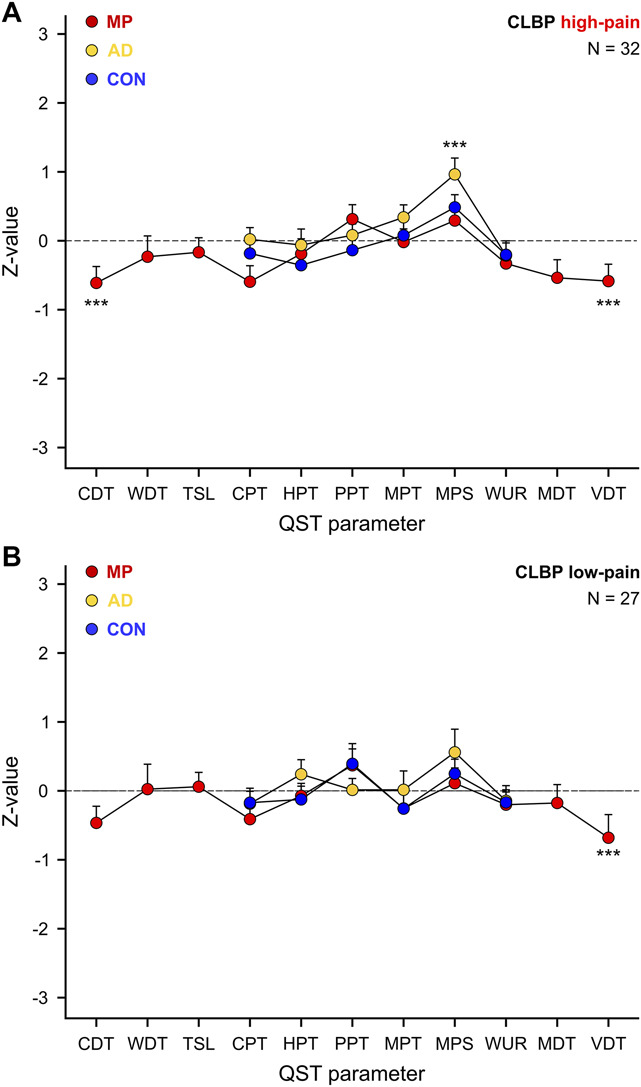
Sensory loss of function in MP and sensory gain of function in AD depends on clinical pain severity. Quantitative sensory testing (QST) profiles of the 3 tested areas, ie, most painful area (MP), pain-free area adjacent to MP (AD), and remote pain-free control area (CON) in a high-pain (clinical pain intensity ≥ NRS 4/10) (A) and a low-pain (clinical pain intensity < NRS 4/10) CLBP subsample (B). Data are presented as mean *Z*-values and SEs. CDT, cold detection threshold; CLBP, chronic low back pain; CPT, cold pain threshold; HPT, heat pain threshold; MDT, mechanical detection threshold; MPS, mechanical pain sensitivity; MPT, mechanical pain threshold; PPT, pressure pain threshold; TSL, thermal sensory limen; VDT, vibration detection threshold; WDT, warm detection threshold; WUR, wind-up ratio. ***P < 0.001.

The results in MP and AD were mainly driven by the high-pain (NRS ≥ 4/10) CLBP subsample (Table 3, Fig. 3).

**Table 3:** Quantitative sensory testing measures in high-pain and low-pain chronic low back pain subsamples.

	High-pain CLBP subsample Clinical pain intensity ≥ NRS 4/10 (n = 32)				Low-pain CLBP subsample Clinical pain intensity < NRS 4/10 (n = 27)			
	Raw values*	Z-scores	Z statistic	*P* †	Raw values*	Z-scores	Z statistic	*P* †
MP‡								
CDT [°C]	**2.9 (2.25)**	−**0.6 (1.35)**	−**3.5**	**<0.001**	3.0 (2.04)	−0.5 (1.26)	−2.4	0.016
WDT [°C]	3.4 (1.73)	−0.2 (1.71)	−1.3	0.190	3.1 (1.15)	0.0 (1.88)	0.1	0.898
TSL [°C]	6.9 (3.39)	−0.2 (1.19)	−0.9	0.345	6.6 (2.56)	0.1 (1.08)	0.3	0.754
CPT [°C]	11.6 (10.73)	−0.6 (1.32)	−3.4	0.002§	12.2 (10.1)	−0.4 (1.19)	−2.1	0.099§
HPT [°C]	43.2 (4.10)	−0.2 (1.23)	−1.1	0.428§	42.9 (3.11)	−0.1 (0.94)	−0.4	0.708§
PPT [kg/cm^2^]	5.3 (2.77)	0.3 (1.18)	1.8	0.226§	5.3 (2.57)	0.4 (1.24)	1.9	0.081§
MPT [mN]	31.0 (34.12)	−0.0 (1.08)	−0.1	0.917§	36.3 (40.22)	−0.3 (1.35)	−1.3	0.278§
MPS [NRS]	4.9 (4.59)	0.3 (1.02)	1.7	0.097§	4.6 (6.43)	0.1 (1.15)	0.6	0.563§
WUR [NRS ratio]	3.0 (2.00)	−0.3 (0.91)	−1.9	0.186§	3.1 (1.98)	−0.2 (0.96)	−1.0	0.452§
MDT [mN]	20.3 (37.18)	−0.5 (1.48)	−3.0	0.002	12.9 (23.66)	−0.2 (1.39)	−0.9	0.361
VDT [V.U.]	**5.7 (2.33)**	−**0.6 (1.39)**	−**3.3**	**<0.001**	**5.2 (2.95)**	−**0.7 (1.75)**	−**3.5**	**<0.001**
PHS [count]	0			0.381	2			1¶
DMA [count]	**9**			**0.033** §	4			1§¶
AD‡								
CPT [°C]	11.7 (10.56)	0.0 (0.96)	0.1	0.906§	8.9 (10.80)	−0.2 (0.93)	−1.0	0.373§
HPT [°C]	43.7 (3.98)	−0.1 (1.32)	−0.4	0.726§	42.7 (3.54)	0.2 (1.10)	1.3	0.634§
PPT [kg/cm^2^]	5.6 (2.41)	0.1 (0.91)	0.5	0.647§	5.9 (2.43)	0.0 (0.86)	0.1	0.942§
MPT [mN]	38.9 (41.8)	0.3 (1.02)	1.9	0.163§	52.6 (90.06)	0.0 (1.42)	0.1	0.938§
MPS [NRS]	**4.2 (4.12)**	**1.0 (1.34)**	**5.5**	**<0.001** §	3.7 (3.83)	0.6 (1.74)	2.9	0.011§
WUR [NRS ratio]‖	3.0 (1.66)	−0.2 (1.01)	−1.2	0.252§	3.2 (2.44)	−0.1 (1.15)	−0.8	0.452§
DMA [count]	8			0.381§	5			0.333§¶
CON								
CPT [°C]	9.0 (9.94)	−0.2 (1.22)	−1.0	0.450§	8.8 (8.72)	−0.2 (1.09)	−0.9	0.373§
HPT [°C]	45.5 (3.80)	−0.4 (1.16)	−2.0	0.135§	44.7 (3.46)	−0.1 (1.00)	−0.6	0.708§
PPT [kg/cm^2^]	4.5 (2.15)	−0.1 (1.14)	−0.8	0.647§	4.0 (1.94)	0.4 (1.52)	2.0	0.081§
MPT [mN]	52.7 (69.39)	0.1 (1.48)	0.4	0.917§	56.6 (69.97)	−0.3 (1.33)	−1.3	0.278§
MPS [NRS]	4.9 (5.01)	0.5 (1.04)	2.7	0.009§	4.5 (6.41)	0.2 (1.09)	1.3	0.293§
WUR [NRS ratio]	4.2 (11.33)	−0.2 (1.14)	−1.1	0.252§	2.4 (1.36)	−0.2 (0.95)	−0.9	0.452§
DMA [count]	7			0.125§	**3**			0.667§¶

All QST measures except for CPT, HPT, and VDT were log-transformed before *Z*-score conversion. Values are presented as mean (SD). Minimum and maximum *Z*-score value for each QST measure was set to ±4 to reduce influences of single extreme outliers. Effect sizes are not reported because for *Z*-tests against mean = 0 and SD = 1, Cohen's *d* is identical to the mean of the *Z*-scores.

* Raw values might not reflect *Z*-scores because (1) most QST measures were log-transformed before *Z*-score conversion, (2) outlier influences are larger for raw values than for *Z*-scores, and (3) raw values are not age and sex matched.

† Statistical comparison to an ideal *Z*-value distribution (mean = 0, SD = 1) for *Z*-tests and to control participants for Fisher's exact tests.

‡ N = 35 for control participants because 10 control participants had been matched to another chronic pain cohort of the Clinical Research Priority Program “Pain.”

§ FDR-corrected for three tests.

‖ N = 31 for high-pain CLBP subsample because 1 patient rated the highest possible single stimulus intensity as not painful.

¶ Fisher's exact test.

AD, pain-free area adjacent to most painful area; CDT, cold detection threshold; CLBP, chronic low back pain; CON, remote, pain-free control area; CPT, cold pain threshold; DMA, dynamic mechanical allodynia; FDR, false discovery rate; HPT, heat pain threshold; MDT, mechanical detection threshold; MPS, mechanical pain sensitivity; MPT, mechanical pain threshold; NRS, numeric rating scale; PHS, paradoxical heat sensation; PPT, pressure pain threshold; QST, quantitative sensory testing; TSL, thermal sensory limen; VDT, vibration detection thresholds; V.U., vibration units in X/8; WUR, wind-up ratio. Bold entries: *P* < 0.05 for PHS and DMA and *P* < 0.001 for other QST measures.

Two significant associations (out of 30 tested) between QST measures indicative of spinal or supraspinal sensitization and patient-reported outcome measures supposedly associated with central pain processes were identified. First, patients with lower PPTs in AD, reflecting more pronounced pressure pain sensory gain of function, reported more widespread pain (rho = 0.31, *P* = 0.017). Second, patients with lower CPTs in CON, reflecting more pronounced cold pain sensory loss of function, had higher PSQ-3 scores, meaning worse sleep quality (rho = −0.30, *P* = 0.022). The remaining associations were not significant (PCS: all rhos < |0.18|, all *P*s > 0.171; PSQ-3: all rhos < |0.18|, all *P*s > 0.182; WPI: all rhos < |0.19|, all *P*s > 0.150).

Influences of pain-relevant medication intake on QST measures were not analyzed because regular intake was reported by less than a quarter (ie, 14/59) of the patients with CLBP, resulting in low statistical power for a respective subgroup analysis.

### 3.3. Clinical examination

The patients with CLBP predominantly presented with features of nociceptive pain mechanisms^51^ (Table 1).

## 4. Discussion

Using QST in 3 different body areas, this study demonstrated area-specific sensory alterations in patients with nonspecific CLBP compared with pain-free control participants, namely: (1) hypoesthesia in the painful MP, (2) mechanical hyperalgesia and allodynia in the MP-surrounding pain-free AD, and (3) no sensory alterations in the remote, pain-free CON. The sensory alterations were more pronounced in patients with clinically more severe CLBP and indicate an involvement of spinal sensitization in CLBP without evidence for peripheral and supraspinal sensitization. No compelling evidence was found for an association between QST measures indicative of spinal or supraspinal sensitization and pain catastrophizing, sleep quality, or widespread pain.

Tactile hypoesthesia in painful body areas has been previously observed in chronic myofascial pain,^18,38^ muscle pain,^24^ and over trigger points,^2^ which supports a muscular/myofascial (Table 1) component in the present CLBP cohort. The findings further align with reports of decreased tactile acuity in the lower back of patients with CLBP.^33,37,40^ Also, despite predominant nociceptive pain features in the investigated CLBP cohort, the observed hypoesthesia in MP could indicate an additional neuropathic component because hypoesthesia is a hallmark of nerve damage.^32^ Neuropathic pain mechanisms have been suggested to play a role in CLBP.^6^ Peripheral fiber loss could also preclude the detection of hypersensitivity in the affected area^50^ and thus explain the findings in MP, particularly the absence of deep tissue hypersensitivity. Alternatively, sensory attenuation could result from neglect-like tactile dysfunction^39^ or enhanced descending pain inhibition, which has been shown to reduce primary hyperalgesia.^58^ Interestingly, 2 and 4 extreme *Z*-score values below −4 (and none above 4) were identified for CDT and VDT in MP, respectively, and adjusted to a value of −4 to minimize outlier influences on the analysis. This means that the sensory loss in MP was even more pronounced in the CLBP cohort and that the reported effect size is a conservative estimate of the true group difference. The lack of a statistically significant deep tissue hypersensitivity in form of decreased PPT values was unexpected based on previous studies in patients with low back pain.^12^ Nevertheless, before multiple comparison correction, a trend (*P* = 0.009) for decreased PPTs had been observed in MP for patients with CLBP. Besides the mechanisms outlined above, the nonsignificant result for PPTs in MP could also be due to the included CLBP patient cohort. The patients were psychologically relatively mildly affected, as evident in lower HADS and PCS scores compared with other CLBP cohorts^19,21,55,62,63^ and a small proportion reporting regular pain-relevant medication intake.

Given that AD was located adjacent to MP and thus, innervated by adjacent spinal segments, sensory gain of function in AD indicates sensitization at the spinal level. Particularly, the sensory gain of function was restricted to superficial mechanical stimuli, aligning with sensory signs observed in areas of secondary hyperalgesia.^31,34,48^ Of note, different spinal sensitization mechanisms have to be considered depending on the location of AD. The originally described activity-dependent central sensitization at the spinal dorsal horn neuron results in ipsilateral hypersensitivity within the receptive field of the same spinal dorsal horn neuron.^65^ By contrast, glia-mediated spinal sensitization has been shown to spread along the spinal cord^8,30^ and can induce widespread sensitization and hypersensitivity. For patients with MP in the midline and thus, an unclear attribution of AD to the ipsilateral or contralateral body side, activity-dependent and glia-mediated sensitization are possible. For patients with lateralized MP, AD was always assessed contralaterally, and therefore, glia-mediated spinal sensitization is more plausible. In the present study, most patients presented with a lateralized MP. However, according to the obtained pain drawings, their pain frequently (52.3%) extended beyond the midline, making it impossible to identify the side of the spinal cord that innervated MP and thus might have been affected by activity-dependent central sensitization. In addition, MP was used as best available proxy for the primarily affected area, but it does not necessarily reflect the location of a primary nociceptive driver. Finally, similarly to MP, the *Z*-score adjustment of extreme outlier values in AD led to a slight underestimation of the sensory gain in patients with CLBP, given that 1 MPS and 1 DMA value were adjusted from values above 4 to 4.

Evidence regarding widespread hyperalgesia in CLBP is inconclusive,^46^ with various studies not reporting sensory alterations in remote body areas of patients with CLBP.^15,27,36,43^ The absence of widespread hyperalgesia in the present cohort might be because of most patients showing localized pain^20^ (Table 1). This feature is considered an indicator of nociceptive pain mechanisms that are not expected to be associated with generalized pain hypersensitivity.^51^ The notion of more widespread clinical pain being associated with experimental pain hypersensitivity beyond the primarily affected area may be reflected in the correlation between higher WPI scores and higher pressure pain sensitivity in AD observed in the present study. However, this correlation might represent a spurious finding because of its small effect size and because it was one of 2 significant correlations out of 30 exploratory correlation analyses, a proportion of positive findings expected by pure chance. The predominance of nociceptive pain mechanisms in the investigated patients with CLBP is further supported by the lower HADS and PCS scores compared with other CLBP studies.^21,62,63^ According to expert consensus, the absence of significant psychological features supports the dominance of nociceptive or neuropathic pain mechanisms.^51^ Interestingly, PCS scores were not associated with QST measures indicative of spinal or supraspinal sensitization. This means that the here observed signs of spinal sensitization may be independent of psychological features and might mainly represent a biological phenomenon. To the best of our knowledge, the present study is the first to allow the differentiation between spinal and supraspinal sensitization in nonspecific CLBP based on area-specific sensory alterations.

The observed hypoesthesia in MP and the mechanical hypersensitivity in AD were driven by the high-pain CLBP subsample, adding to previous work in chronic pain cohorts, which showed that clinical pain severity varied across detected QST profiles.^16,25,41^ Thus, in combination with other factors, clinical pain intensity can be an indicator of pathophysiological mechanism in CLBP. Alternatively, central sensitization might cause more severe CLBP.^47^ Furthermore, the high-pain CLBP subsample showed higher PSQ-3 scores compared with the low-pain CLBP subsample. This suggests that poorer sleep quality could play a role in the sensory alterations observed in the high-pain CLBP subsample. Yet, the sleep impairment (median PSQ-3 score: 99) was less pronounced compared with previous reports (mean PSQ-3 score: 187.2)^4^ and the bidirectional relationship between pain intensity and sleep quality^1^ makes it challenging to determine which of the 2 is more relevant in the context of the present results. A generic association of poor sleep quality with signs of spinal or supraspinal sensitization is not supported by the present study because PSQ-3 scores were not associated with any sensory gain of function in AD or CON, respectively. The correlation between PSQ-3 and more pronounced sensory loss of function, ie, lower CPTs in CON, might, as mentioned above for the correlation between WPI and PPTs in AD, be a spurious finding as the effect size was low and the exploratory correlation analyses were not corrected for multiple comparisons.

One limitation of the present study is that a reduced QST protocol was used in AD and CON, including the consequence that MPS and DMA were calculated based on only 2 stimulus-response-function blocks instead of 5. The reduced QST protocol precluded the detection of potential sensory loss in AD and CON. For CON, sensory loss was unlikely given that the hand was sensory intact in all participants (as assessed by bedside sensory testing). However, sensory loss in AD cannot be ruled out. In addition, the differences between the applied QST protocol and the full DFNS QST protocol hamper the comparability of the absolute QST values between this study and studies that used the full DFNS QST protocol. Nevertheless, the presented relative comparison of patients with CLBP and control participants is valid because the identical QST protocol had been used in both cohorts. Furthermore, the low proportion of patients with CLBP reporting regular pain-relevant medication intake prevented a meaningful analysis of whether medication had an influence on QST measures. Yet, the small number of patients with CLBP relying on regular medication intake also represents a strength of the present study. First, there are fewer confounding medication effects. Second, it might indicate that the included CLBP cohort was relatively mildly affected by their pain with a lesser role of psychosocial factors,^26^ highlighting the potential relevance of the study's findings from a biological perspective. Finally, it should be kept in mind that QST only allows an indirect assessment of central sensitization as defined by the International Association for the Study of Pain^28^ by sensory proxies. Alternative methods might more closely reflect changes in neuronal hyperexcitability, for example, the N13 component of somatosensory evoked potentials.^14^ However, by considering known manifestations of peripheral, spinal, and supraspinal sensitization, QST can be used to infer the presence of central sensitization, if assessed in a primarily affected, secondary, *and* remote body area.

In conclusion, the combination of QST in 3 different body areas allowed to investigate contributions of peripheral, spinal, and supraspinal sensitization in patients with CLBP. Signs of spinal sensitization were observed in patients with CLBP, predominantly in patients with more severe clinical pain who also displayed poorer sleep quality compared with less severely affected patients. Of particular interest, the included patients with CLBP presented with predominant nociceptive features, namely localized pain and a low degree of psychological interference, and the observed signs of spinal sensitization were independent of pain catastrophizing levels. The present study might thus offer insights into central pain processes involved in nonspecific, nociceptive CLBP phenotypes without pronounced influence of psychological factors.

## Disclosures

The authors have no conflict of interest to declare.
